# Dr. Hutchinson on Popliteal Aneurism

**Published:** 1812-03

**Authors:** A. Copland Hutchinson

**Affiliations:** Royal Naval Hospital, Deal


### Medical and Physical Journal.

3 OF VOL. XXVII.] MARCH, 1812. [NO. 157.

*To the Editors of the Medical and Physical Journal.*

GENTLEMEN,

IN hopes the accompanying case of Popliteal Aneurism, in which the femoral artery has been a third time successfully tied, from the outer margin of the sartorius muscle, will not be unacceptable to your chirurgical readers, I send it for insertion in your valuable Journal.

I am, Gentlemen,

Your most obedient Servant,

A. COPLAND HUTCHINSON, M.D.

*Royal Naval Hospital, Deal,*

*3d January,* 1812.

Fred. Wilkie, a Prussian, aged 36, of a spare and lax fibre, was admitted a patient under my care from his Majesty's ship Poictiers, on the 18th of November, 1811, for cure of Popliteal Aneurism.

No evident or immediate cause could be assigned for the production of the disease. The aneurismal tumor appeared on the 3d of November. A few days previous to this he had a slight degree of pain in the ham, which precluded a free exercise of the limb. By the patient's own account, however, he has been very subject to pains in the gastrocnemius muscle of the affected leg, extending upwards to the condyles of the femur, ever since the precipitate and harassing retreat of the Prussian troops from the banks of the Elbe, in 1804, in which service he was then a soldier, acting in the rifle corps.

At his admission into the hospital, the tumor pulsated strongly, and pushed out the flexor tendons, forming the ham-strings, so considerably, that he could not extend or contract the leg one degree from a right angle, which it formed with the thigh—the pain occasioned by the attempt was so excruciating.

The tumor continued rapidly to increase, up to the day the operation was performed, when its pulsations had become obscure, and the integuments discolored over nearly the whole of its surface. There was no œdema of the foot or ankle, and but a little over the upper part of the tibia. His bowels were opened, twelve ounces of blood abstracted from the arm on the 3d of December, and on the 4th the femoral femoral artery was tied with the utmost facility from the outer margin of the sartorius muscle, about an inch higher than where it pierces the tendon of the triceps.

From this period to the day of his discharge, the patient was free from every species of complaint, and there was not any diminution of heat observable in the limb from the completion of the operation. The ligatures were removed on the fourteenth and eighteenth days, and the wound was completely cicatrised on the twentieth from the operation.

I do not recollect to have met, in the course of my reading or observation, a case of aneurism wherein the disease increased so rapidly as in that before us. The aneurismal tumor had attained so great a size, and the integuments covering it from one ham-string to the other were so highly discolored, that I am strongly inclined to believe the tumor would have burst had the operation been postponed only a few days longer.

The facts detailed in the present case, in addition to those already submitted to the public, in a pamphlet lately published by me, are such, I think, as tend firmly to establish the great, if not superior, advantages to be derived from securing the femoral artery by the outer margin of the sartorius, in place of the inner margin of that muscle, as heretofore practised since the days of the celebrated John Hunter.

In Wilkie's case, the sartorius, when raised and supported by the finger instrument, continued as quiescent as if possessed of no living principle. The dissection was not impeded, nor the parts obscured, by any effusion of blood during the operation. The wound was not kept open by a discharge of lymph from the division of lymphatic vessels; there were no collections of matter formed under the muscle to retard the cure; and the whole was completely cicatrised within three weeks, and the patient discharged to his ship before the expiration of a month; at which period the limb was as much restored to its customary functions as the remains of the tumor would admit.

The relation of this case tends likewise to confirm the opinions entertained by Mr. John Bell, of Edinburgh, and Mr. Ramsden, of London, that delay in performing the operation for aneurism, with the view of giving time for the dilatation of anastomosing vessels, is perfectly unnecessary; and, if pushed to any length, may be attended by the most distressing consequences.

Mr. Astley Cooper, to whom our profession is so much indebted, observes, that it is not until the full expiration of twelve months, after the femoral artery has been tied for popliteal popliteal aneurism, that the obliteration of this vessel, up to the arteria profunda, is found to be complete.* The muscular branches, therefore, between the ligature and where the profunda goes off, must be highly serviceable in contributing to the nourishment of the limb below, during the early part of this period ; a period in which the circulation is most languid.

The operation for popliteal aneurism, as it is generally performed, is so high up in the thigh, that, if secondary hæmorrhage were to occur, there is scarcely space enough below Pouparts ligament to tie the artery again, nor can amputation be performed with much prospect of success ; and, should the external iliac be exposed and secured, there are few of us, I am convinced, who would augur so favorably of the termination of the case, as when the artery is tied on the forepart of the thigh, or the limb removed by amputation.

The principles, therefore, upon which I ventured to recommend the operation for popliteal aneurism to be performed lower down on the forepart of the thigh, and from the outer margin of the sartorius, do not appear to me to be in the slightest degree invalidated by the result of the late ingenious experiments, or by what has recently been written on the subject: on the contrary, every case that has fallen under my personal observation, and all the reflection I have been able to bestow on this interesting topic, have served only to establish more firmly in my mind the propriety, utility, and soundness, of the doctrine I have labored to inculcate.

* See this gentleman's valuable Paper in the 2d vol. of the Medico-Chirurgical Transactions.

